# Leadless pacemaker in atrioventricular block patients with specific indications: a systematic review

**DOI:** 10.3389/fcvm.2026.1910998

**Published:** 2026-09-14

**Authors:** Lingling Wang, Liang Ning, Zhuoga Lamu, Shuanshan Ren, Jinzhou Xie, Liangzhang He, Yufeng Li

**Affiliations:** 1 School of Social Work and Health Management, Xihua University, Chengdu, China; 2 Department of Cardiovascular, 363Hospital, Chengdu, Sichuan, China

**Keywords:** atrioventricular block, complications, leadless pacemaker, systematic review, traditional intravenous pacemakers

## Abstract

**Background:**

Leadless cardiac pacemakers are becoming a viable alternative to traditional intravenous pacemakers. This study aims to systematically evaluate the safety of using leadless pacemakers in patients with atrioventricular block (AVB) who meet specific indications, based on existing published research.

**Methods:**

A comprehensive literature search was conducted in PubMed, Web of Science, CNKI, Embase, Cochrane Library, ClinicalTrials.gov, WHO International Clinical Trials Registry Platform (ICTRP) and Wanfang Database for clinical studies on leadless pacemaker treatment for atrioventricular block. Data analysis was conducted using RevMan and R software. Relative risk (RR) with 95% confidence interval (CI) was calculated. Heterogeneity was assessed using the I^2^ statistic.

**Results:**

A total of 60 studies were ultimately included, with AVB patients accounting for 6% to 100% of the implanted population. The overall incidence of adverse events in patients receiving leadless pacemakers was low. Among these, 16 studies provided complication data for the AVB subgroup and were included in the meta-analysis. The results showed that, compared with the control group, leadless pacemakers did not significantly reduce the risk of adverse events in AVB patients (RR = 1.41, 95% CI: 0.43–4.60, *P* = 0.57). Subgroup analysis by device type demonstrated no statistically significant differences in adverse event risks compared to traditional pacemakers for either single-chamber ventricular leadless pacemakers (RR = 0.26, 95% CI: 0.03–2.01, *P* = 0.20) or AV-synchronous/dual-chamber leadless pacemakers (RR = 2.16, 95% CI: 0.76–6.17, *P* = 0.15), with no significant interaction between subgroups. In the single-arm rate analysis, five cohort studies reported an adverse event rate of 14% (95% CI: 0.04–0.24); after applying the Freeman-Tukey transformation to eleven single-arm studies, the adverse event rate was 1.15% (95% CI: 0.26%–2.67%).

**Conclusion:**

In patients with atrioventricular block, leadless pacemakers demonstrate no significant difference in the risk of adverse events compared with conventional pacemakers, with lower complication rates and favorable clinical feasibility.

## 1 Introduction

Atrioventricular block (AVB) is defined as a delay or interruption in the transmission of an impulse from the atria to the ventricles. AVB is classified according to the severity of the conduction disturbance. In first-degree AVB, all atrial impulses are conducted to the ventricles, but the conduction time is prolonged. In second-degree AVB, some atrial impulses are conducted to the ventricles and some are not. In third-degree AVB, no atrial impulses are conducted to the ventricles (1). Treatment of AVB aims at ameliorating symptoms and preventing syncope and sudden cardiac death (SCD). Syncope and dizziness are mainly observed in high-degree and complete AVB, especially in the paroxysmal forms. Death in patients with untreated AVB is due not only to heart failure secondary to low cardiac output, but also to SCD caused by prolonged asystole or bradycardia-triggered ventricular tachyarrhythmia. Therefore, high-degree and complete AVB are associated with syncope and SCD, underscoring their life-threatening potential (2).

Currently, the treatment strategy for AVB is individualized based primarily on the degree of block and the presence of clinical symptoms. In patients with acquired second-degree Mobitz type II AVB, high-grade AVB, or third-degree AVB not attributable to reversible or physiologic causes, permanent pacing is recommended regardless of symptoms (3). Beyond conventional transvenous pacing systems, leadless pacemakers represent a major technological breakthrough in recent years, with their indications gradually being refined in eligible patients. According to the 2021 ESC Guidelines on cardiac pacing and cardiac resynchronization therapy, leadless pacemakers should be considered as an alternative to transvenous pacemakers when no upper extremity venous access exists or when the risk of device pocket infection is particularly high, such as previous infection and patients on haemodialysis. Furthermore, taking into consideration life expectancy and using shared decision-making, leadless pacemakers may be considered as an alternative to standard single-lead ventricular pacing (2). Leadless pacemakers are broadly classified into two types according to pacing mode and sensing function: single-chamber ventricular pacemakers and atrioventricular (AV) synchronous/dual-chamber pacemakers. The former operate in the VVI (Ventricular Pacing, Ventricular Sensing, Inhibited Response) mode, with the electrode placed in the right ventricle to sense intrinsic ventricular activity and deliver pacing pulses on demand, lacking both atrial sensing and AV sequential contraction. The latter, however, use refined sensing algorithms to achieve AV synchrony, which sense atrial electrical activity and trigger ventricular pacing, thereby mimicking physiological AV sequential contraction to preserve ventricular filling and cardiac output.

In current clinical practice, leadless pacemakers have accumulated substantial real-world experience. Multiple meta-analyses have compared their clinical outcomes with those of conventional transvenous pacemakers, and the results have shown that leadless pacemakers are associated with a lower overall complication rate than traditional pacing systems (4–7). The most recent meta-analysis was published online in December 2025 and in print in March 2026, with a systematic literature search conducted up to October 2024 (7). However, existing studies have largely focused on overall outcomes in mixed populations encompassing various indications, and dedicated subgroup analyses specifically targeting patients with AVB are lacking. Consequently, the findings from these studies may obscure potential differences in safety profiles across etiologic subgroups. To address this gap, the present study focuses on the safety of leadless pacemakers specifically in patients with AVB. Unlike previous studies involving heterogeneous populations, this study exclusively enrolls a patient cohort with a confirmed diagnosis of AVB and strictly followed the PICO framework as follows: *P* (Population) was defined as high-degree or complete atrioventricular block; I (Intervention) refers to acceptance of a leadless pacemaker implantation; C (Comparator) is a paired cohort receiving traditional transvenous single- or dual-chamber pacemaker implantation during the same period; O (Outcomes) are safety indicators. The aim of this study is to provide more accurate and targeted evidence-based support for the safety of leadless pacing technology in AVB patients by comparing perioperative and long-term major adverse events between leadless pacemakers and traditional pacemakers in this specific population.

## 2 Methods

### 2.1 Literature searching

This systematic review was conducted and reported in accordance with the Preferred Reporting Items for Systematic Reviews and Meta-Analyses (PRISMA) 2020 Statement. A completed PRISMA 2020 checklist is provided as Supplementary Table S1. This review was not prospectively registered in PROSPERO or any other international registry. A comprehensive literature search was performed in PubMed, Embase, Web of Science Core Collection, the Cochrane Library (CENTRAL), China National Knowledge Infrastructure (CNKI), Wanfang Database, ClinicalTrials.gov, and the World Health Organization International Clinical Trials Registry Platform (WHO ICTRP) from database inception to January 2026. Studies published in English or Chinese were considered eligible.

The search strategy combined controlled vocabulary [Medical Subject Headings (MeSH) in PubMed and Emtree terms in Embase, where applicable] with free-text terms using Boolean operators. Search terms included “leadless pacemaker,” “leadless pacing,” “leadless cardiac pacemaker,” “Micra,” “Micra TPS,” “Micra AV,” “Micra AV2,” “Nanostim,” “AVEIR,” “AVEIR VR,” “AVEIR DR,” “atrioventricular block,” “atrioventricular conduction block,” “AV block,” and “AVB.” Corresponding Chinese search terms were used for CNKI and Wanfang Database. The detailed search strategies for each database are provided in Supplementary Table S2. All retrieved records were imported into EndNote X9 for duplicate removal before title/abstract screening and full-text review.

### 2.2 Inclusion and exclusion criteria

Inclusion criteria included: (1) Patients clinically diagnosed with AVB who underwent leadless pacemaker implantation were included; (2) The study types included randomized controlled trials, non-randomized controlled trials, cohort studies, or single-arm studies; (3) The outcomes of interest were reported in the study, and reliable data on the required endpoints could be extracted from the full text. Exclusion criteria included: (1) The definition of outcome indicators is vague and there is no research that provides real data; (2) Repeated reporting, incomplete data information, and inability to obtain full-text research; (3) The literature forms include reviews, animal experiments, systematic reviews, and case studies.

### 2.3 Data extraction

Two researchers independently screened the literature, extracted the data, and cross-checked the results. Any disagreements were resolved through discussion. The extracted data encompassed: (1) fundamental details about the enrolled studies, such as the first author's name, publication year, and study type; (2) general characteristics of the study population, including the device type, sample size, type of AVB, age distribution, and follow-up duration; (3) specified outcomes of interest.

### 2.4 Endpoints

The primary endpoint of this study was the occurrence of adverse events, including cardiac tamponade/pericardial perforation, access site complications (hematoma, arteriovenous fistula, pseudoaneurysm), chronic pacing threshold elevation, pacing-induced cardiomyopathy, pacemaker syndrome, infection, lead dislodgement, and other lead/device-related events.

All adverse events were temporally stratified based on prespecified cut-off values: short-term complications were defined as any adverse events occurring from the time of implantation to within 30 days post-procedure or prior to hospital discharge, whichever occurred later, primarily including cardiac tamponade/perforation, acute vascular injury, access-site hematoma, and early lead or device dislodgement; whereas long-term complications were defined as any adverse events occurring beyond 30 days post-implantation, encompassing pacing-induced cardiomyopathy, late-onset device infection, and reinterventions necessitated by premature battery depletion or system malfunction.

### 2.5 Quality assessments

The methodological quality of the included observational cohort studies was assessed using the Newcastle-Ottawa Scale (NOS). This scale has a maximum score of 9 points, and studies with a total score of ≥7 points were considered to be of high quality. For single-arm studies, the corresponding critical appraisal tool developed by the Joanna Briggs Institute (JBI) was used. This tool consists of 10 items and evaluates the quality of the included studies from the aspects of case selection, ascertainment of the disease or health problem, and presentation of case data. Each item is rated as “yes,” “no,” “unclear,” or “not applicable.”

### 2.6 Statistical analysis

The statistical analysis of this study was conducted using the ReviewManager 5.4 software provided by Cochrane. The outcome measure is a binary variable, and interval estimation is performed using a 95% confidence interval (CI). The effect size is represented by the RR value to determine whether the result is statistically significant. The *P*-value is used as the standard, with *P* < 0.05 indicating statistical significance and *P* ≥ 0.05 indicating no statistical significance. Using the *I*^2^ test to evaluate heterogeneity between studies, when *P* > 0.1 and *I*^2^ < 50%, it can be considered that there is no significant statistical heterogeneity between multiple studies, and a fixed effects model is used for meta-analysis; When *P* ≤ 0.1 and *I*^2^ ≥ 50%, it can be considered that there is significant statistical heterogeneity between multiple studies, and further subgroup analysis is performed. Meta analysis uses a random effects model.

## 3 Results

### 3.1 Literature screening process and results

By searching four databases at home and abroad, a total of 2,723 articles were obtained. After removing 280 duplicate articles, the remaining number of articles was 2,433. After preliminary reading of the title and abstract of the paper, a total of 2,344 articles were screened out, including meta-analysis, expert opinions, reviews, case reports, and research content that did not match, leaving a total of 99 articles. Further reading of the full text excluded 39 articles that could not extract data and did not meet the inclusion criteria, resulting in 60 articles being included in the meta-analysis (8–24). The process of literature search and screening is shown in Figure 1.

**Figure 1:**
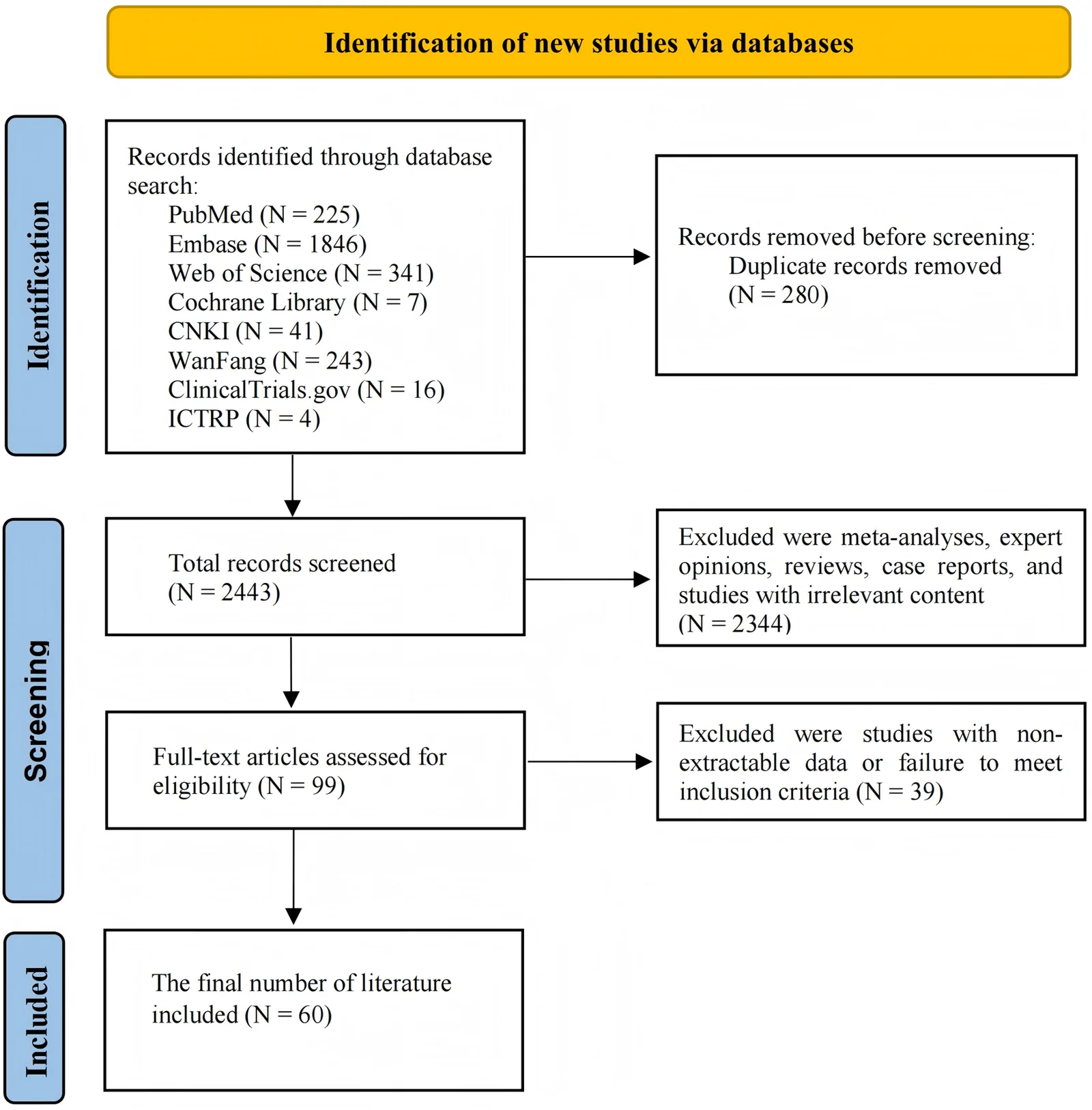
PRISMA flow diagram of the literature search.

### 3.2 Patient characteristics

A total of 60 articles were included in this study. The extracted baseline characteristics comprised publication years, study type, average age, sample size, follow-up time, AVB type, device type, and implantation success rate. The included research designs consisted cohort studies and single arm studies. Within the leadless pacemaker group, the implanted devices included Micra, Aveir DR, and Nanostim. The baseline characteristics of the cohort studies were shown in Table 1, While those of single-arm clinical studies were detailed in Table 2.

**Table 1:** Basic characteristics of included studies (cohort studies, *n* = 20).

Study	Design	Average age(y)		Totalsamplesize (*n*)		Pacemakertype	
		Experimental	Control	Experimental	Control	Experimental	Control
Biffi et al., 2025 (8)	Cohort study	72.2 ± 13.6	74.9	22	66	Micra AV	RVP, HBP
Katsuki et al., 2022 (12)	Cohort study	84.7 ± 7.1	77 ± 9.5	368	561	Micra VR	PM
Sasaki et al., 2022 (13)	Cohort study	81 ± 8	82 ± 6	58	58	VVI-LPM, Micra VR	DDD-TPM
Lenormand et al., 2023 (9)	Cohort study	77 ± 8	80 ± 8	48	45	Micra AV	Micra VR
Markson et al., 2025 (14)	Cohort study	74 ± 15	75 ± 13	2594	2594	LPM	PM
Chopra et al., 2025 (10)	Cohort study	77.2 ± 11.6	74.7 ± 13.4	25	70	Micra AV	LBBAP
Mararenko et al., 2023 (15)	Cohort study	78.28	81.71	4,105	17,677	LPM	PM
Schiavone et al., 2023 (16)	Cohort study	78.9	77.0	72	1,082	LPM	LPM
Garweg et al., 2024 (17)	Cohort study	74.1	71.1	801	2,667	Micra AV	LPM
Russo et al., 2022 (18)	Cohort study	75.5	78.1	73	67	Micra VR	Micra VR
Ma et al., 2025 (19)	Cohort study	71 ± 14	64 ± 12	20	16	Micra (low-septal)	Micra (mid-septal)
Fouad et al., 2025 (20)	Cohort study	74.9	77.4	1,158	1,158	LPM	TV-VVI
Togashi et al., 2023 (11)	Cohort study	85 ± 10	85 ± 5.9	17	22	VVI-LPM	DDD-TPM
Palmisano et al., 2025 (25)	Cohort study	71.1 ± 16.2	71.8 ± 15.0	189	189	Aveir VR	Micra VR
Garweg et al., 2020 (26)	Cohort study	82.5	82.0	54	116	Micra TPS	Micra TPS
Gao et al., 2023 (27)	Cohort study	78.0	79.0	27	33	Micra TPS	PM
Ze et al., 2022 (28)	Cohort study	69.9 ± 12.2	79.2 ± 14.2	33	18	LPM	LPM
Wang et al., 2026 (29)	Cohort study	83.1 ± 5.6	62.2 ± 10.1	76	64	LPM	LPM
Lam et al., 2023 (30)	Cohort study	84.14 ± 4.19	82.95 ± 4.87	22	22	Micra AV	DDDR
Jelisejevas et al., 2024 (31)	Cohort study	82.7 ± 9.7	81.0 ± 8.6	24	233	Micra TPS	Micra TPS

Study	Patient type distribution	Follow-up duration	Implantation success rate	Specific vascular access	
				Experimental	Control
Biffi et al., 2025 (8)	All patients are AVB	24 months	100%	Femoral	Subclavian
Katsuki et al., 2022 (12)	Overall proportion of AVB patients: 50% (471/929)	1.7 years	100%	Femoral	Not specified
Sasaki et al., 2022 (13)	High-degree AVB accounted for 66%	VVI-LPM group: 801 days; DDD-TPM group: 649 days	100%	Femoral	Not specified
Lenormand et al., 2023 (9)	All patients are AVB	AV group: 8 months; VR group: 20 months	100%	Femoral	Femoral
Markson et al., 2025 (14)	Overall proportion of AVB patients: 12.4% (642/5188)	28.8 months	100%	Femoral	Not specified
Chopra et al., 2025 (10)	All patients are AVB	LBBAP group: 14 ± 8 months; LP group: 13 ± 8 months	100%	Femoral	Axillary
Mararenko et al., 2023 (15)	Second-degree AVB (*n* = 924), third-degree AVB (*n* = 5,859)	30 days	100%	Femoral	Not specified
Schiavone et al., 2023 (16)	Complete AVB: ED + cohort 37.5%, ED- cohort 27.3%	Perioperative period	100%	Femoral	Femoral
Garweg et al., 2024 (17)	High-degree AVB accounted for 55.8% (447/801)	12 months	99.4%	Femoral	Not specified
Russo et al., 2022 (18)	Third-degree AVB: 14.2% (20/140); second-degree AVB: 12.8% (18/140); first-degree AVB: 7.1% (10/140)	Non-AF: 591.1 ± 274.7 days; AF: 620.3 ± 259.1 days	100%	Femoral	Femoral
Ma et al., 2025 (19)	Overall proportion of AVB patients: 66.7% (24/36)	5.6 ± 1.2 months	100%	Femoral	Femoral
Fouad et al., 2025 (20)	AVB accounted for 33.9%	1 week, 6 months, 12 months	100%	Femoral	Not specified
Togashi et al., 2023 (11)	All patients are AVB	2 years	100%	Femoral	Not specified
Palmisano et al., 2025 (25)	Overall proportion of AVB patients: 29.9% (113/378)	30 days	100%	Not specified	Not specified
Garweg et al., 2020 (26)	High-degree AVB accounted for 51.2% (87/170)	12 months	100%	Not specified	Not specified
Gao et al., 2023 (27)	Overall proportion of AVB patients: 96.7% (58/60)	6 months	100%	Not specified	Not specified
Ze et al., 2022 (28)	Overall proportion of AVB patients: 49.0% (25/51)	1 month, 3 months, 12 months	100%	Femoral	Femoral
Wang et al., 2026 (29)	Third-degree AVB: 30.7% (43/140); second-degree AVB: 12.9% (18/140)	24 h, 3 months, 6 months	100%	Femoral	Femoral
Lam et al., 2023 (30)	All patients are AVB	6 months	100%	Femoral	Subclavian, Cephalic, or Axillary vein.
Jelisejevas et al., 2024 (31)	Overall proportion of AVB patients: 28.8% (74/257)	30 days	98.8%	Femoral	Femoral

**Table 2:** Basic characteristics of included studies (single-arm studies, *n* = 40).

Study	Design	Average age (y)	Samplesize (*n*)	Pacemakertype	Patienttypedistribution	Follow-upduration	Implantationsuccessrate
Sharma et al., 2020 (32)	Single-arm study	80 ± 8.5	35	MICRA TPS	High-degree AVB 34.2%	16.8 months	100%
Arps et al., 2023 (33)	Single-arm study	70.1 ± 14.3	54	Micra VR	Complete heart block 44.4%	10 months	100%
Haddai et al., 2025 (34)	Single-arm study	72.2 ± 10.5	175	AVEIR DR	Third-degree 25.7%, second-degree 10.3%, first-degree 0.6%	1 month	99.40%
Knops et al., 2023 (35)	Single-arm study	69.2 ± 13.5	300	Aveir DR	AVB 33.3%	3 months	98.3%
Ritter et al., 2015 (36)	Single-arm study	77.0 ± 10.2	140	Micra TPS	second-degree 5.7% Third-degree 7.9%	1.9 ± 1.8 months	100%
Yan et al., 2024 (37)	Single-arm study	78	94	Micra VR, Micra AV	Third-degree 35.1% second-degree 31.9%	28 days to 1 year	100%
Strik et al., 2023 (21)	Single-arm study	34 ± 8	35	Micra VR	Complete AVB 29%	26 ± 15 months	100%
Zheng et al., 2024 (22)	Single-arm study	68.2 ± 9.7	68	Micra AV	Third-degree AVB 100%	3 months	100%
Deng et al., 2025 (23)	Single-arm study	69.3 ± 13.6	76	Micra AV	AVB 65.8%	4 months	100%
Zhou et al., 2022 (24)	Single-arm study	80.6 ± 8.6	11	Micra VR	Third-degree AVB 27.3%	1 to 6 months	100%
Garweg et al., 2017 (38)	Single-arm study	79.1 ± 9.7	66	Micra TPS	Third-degree 30.3%, second-degree 3.0%	10.4 ± 6.1 months	98.5%
Knops et al., 2025 (39)	Single-arm study	69.2 ± 13.5	300	Aveir DR	Third-degree AVB 33.3%	12 months	98.3%
Reddy et al., 2014 (40)	Single-arm study	77 ± 8	33	Nanostim	AVB 85%	3 months	97%
Da Costa et al., 2016 (41)	Single-arm study	75 ± 10	14	Micra	AVB 92.5%	≥3 months	100%
He et al., 2023 (42)	Single-arm study	90	11	Micra	AVB 36.4%	8 months	100%
Li et al., 2021 (43)	Single-arm study	65.2 ± 11.4	6	Micra VR	Intermittent AVB 33.3%	6 months	100%
Wu et al., 2023 (44)	Single-arm study	68.5 ± 23.9	22	Micra	Third-degree 36.4%, second-degree 9.1%	3 to 31 months	100%
Chen et al., 2024 (45)	Single-arm study	72.6 ± 15.3	1,620	Micra VR 、Micra AV	AVB 40.0%	1, 3, 6, and 12 months	99.8%
Chen et al., 2025 (46)	Single-arm study	68.8 ± 16.9	438	Micra	AVB 34.70%	1 day to 1 year	100%
Liu et al., 2025 (47)	Single-arm study	77.7 ± 9.8	44	Micra	AVB 45.5%	434.7 days	100%
Zhang et al., 2023 (32)	Single-arm study	90.1 ± 3.8	55	Micra	AVB 67.27%	During hospitalization	100%
Kuang et al., 2021 (48)	Single-arm study	73 ± 14	36	Micra	AVB 44.5%	6 ± 3 months	100%
Chinitz et al., 2023 (49)	Single-arm study	77.2 ± 10.8	152	Micra AV	AVB 36.8%	3 months	100%
Doshi et al., 2025 (50)	Single-arm study	70 ± 13	452	AVEIR DR	AVB 30.5%	30 days	98.7%
Knops et al., 2015 (51)	Single-arm study	76 ± 8	31	Nanostim	AVB 83.9%	12 months	100%
Reddy et al., 2015 (52)	Single-arm study	75.8 ± 12.1	526	Nanostim	AVB 64.6%	6.9 ± 4.2 months	95.8%
Garweg et al., 2019 (53)	Single-arm study	83	133	Micra TPS	High-degree AVB 47.4%	13 ± 11 months	100%
Valiton et al., 2019 (54)	Single-arm study	80.3 ± 11.1	92	Micra TPS	AVB 23.9%	12.4 ± 7.4 months	97.8%
Kumar et al., 2021 (55)	Single-arm study	71.71 ± 8.44	28	Micra TPS	Third-degree 21.4%, second-degree 25%	1 week, 1 month, 3 months, 6 months, 3 years.	100%
Bicong et al., 2022 (56)	Single-arm study	71.1 ± 16.6	39	Micra VR	Complete AVB 64.1%	24.8 ± 14.7 months	100%
Mechulan et al., 2022 (57)	Single-arm study	81.2 ± 6.8	20	Micra AV	High-degree AVB 55.0%	1 month	100%
Beccarino et al., 2023 (58)	Single-arm study	77.4 ± 10.5	86	Micra VR、Micra AV	Complete AVB 45.3%, second-degree 11.6%	163 days	100%
Gul et al., 2024 (59)	Single-arm study	68.9 ± 13.7	40	Micra VR、Micra AV	AVB 67.5%	10 months	100%
Oates et al., 2024 (60)	Single-arm study	68 ± 13	100	Micra VR、Micra AV	Complete AVB 9.0%	10.6 months	100%
Sultan et al., 2024 (61)	Single-arm study	79.7 ± 8.6	188	Micra VR	AVB 6.4%	723.4 ± 597.9 days	100%
El-Bokl et al., 2022 (62)	Single-arm study	11.5	11	Micra	AVB 90.9%	22 months	100%
Denman et al., 2018 (63)	Single-arm study	78	79	Micra	AVB 20.3%	355 days	96.2%
Higuchi et al., 2021 (64)	Single-arm study	86	11	Micra	AVB 36.4%	17.2 ± 6.5 months	100%
He et al., 2022 (65)	Single-arm study	70.56 ± 16.39	20	Micra	AVB 35.0%	19.0 ± 7.4 months	100%
Wong et al., 2024 (66)	Single-arm study	16	10	Aveir VR	AVB 60.0%	9 months	100%

### 3.3 Quality assessment results

The NOS scale identified eighteen cohort studies as high-quality and two as moderate-quality, as described in Supplementary Table S3. The 40 single-arm studies were generally of high quality in domains including case diagnosis, inclusion process, and outcome reporting, but lacked clarity on the inclusion of all eligible cases and geographic/sociological background (Supplementary Table S4).

### 3.4 Descriptive comprehensive analysis

A total of 14,224 patients across 60 observational studies were evaluated. Descriptive analysis indicated that leadless pacing was primarily indicated for AVB, particularly the third-degree subtype, emerging as the leading indication across the cohorts. The prevalence of AVB as an indication ranged from 6% to 100% across the included studies. All studies reported total complication rates ranging from 0% to 40%, with most being mild-to-moderate and manageable vascular access-related or cardiac events. While device-specific variations were noted, device-related complications remained rare. Collectively, these findings suggest that leadless pacemaker implantation for AVB is associated with a high procedural success rate, low overall risk of adverse events, and a very low incidence of severe complications.

### 3.5 Meta-analysis results

The main analysis only included 16 studies that reported subgroup data of AVB. Among them, 5 cohort studies used relative risk (RR) for traditional meta-analysis, and 11 single arm studies used double inverse sine transform for single arm rate meta-analysis, calculating the incidence of complications and 95% CI, Heterogeneity was evaluated using *I*^2^ test.

#### 3.5.1 Comparative analysis

Five cohort studies were included in the meta-analysis to compare the risk of adverse events between leadless pacemakers and transvenous pacemakers in patients with AVB (8–11, 30). The analysis evaluated the risks of adverse events, including perioperative complications, chronic threshold elevation, pacing-induced cardiomyopathy, pacemaker syndrome, and infection. Five cohort studies reported the incidence of these events among AVB patients in the intervention and control groups, allowing for the direct calculation of relative risk (RRs) and their 95% confidence intervals (CIs).

Among them, four studies utilized an external controlled design to compare the safety of leadless vs. traditional intravenous pacemakers in patients with AVB (8, 10, 11, 30). Significant heterogeneity tests were observed across the four studies (*χ*^2^ = 7.56, df = 3, *P* = 0.06, I^2^ = 60%), indicating moderate to high heterogeneity between studies. Consequently, a random-effects model was used for pooling the data. The pooled analysis demonstrated no statistically significant difference between the two groups (RR = 1.41; 95% CI: 0.43–4.60; *P* = 0.57), indicating that leadless pacemakers did not significantly reduce the risk of adverse events compared to conventional pacemakers in patients with AVB (Figure 2).

**Figure 2:**
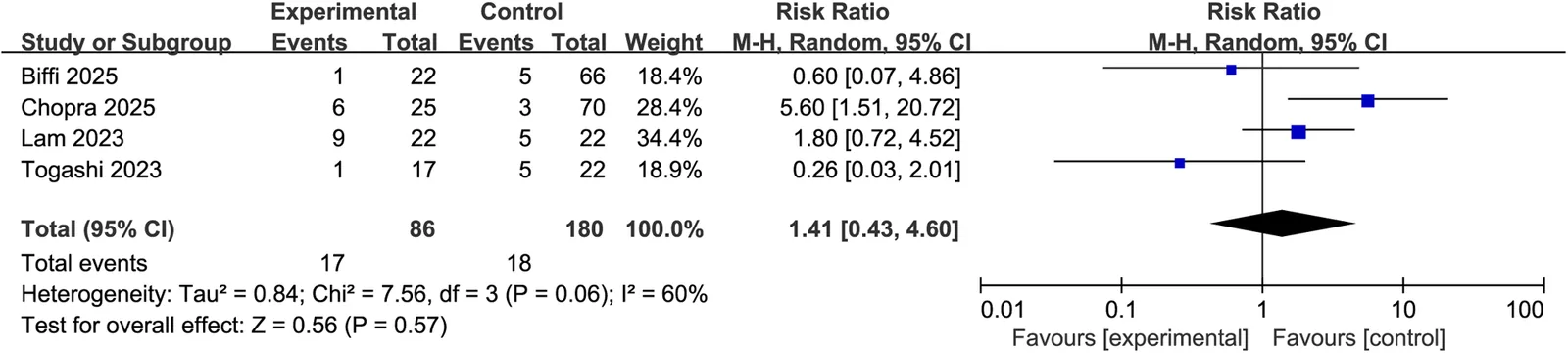
Forest plot of adverse event comparisons between leadless and conventional pacemakers.

To address the substantial heterogeneity observed in the primary analysis (I^2^ = 60%, *P* = 0.06), a subgroup analysis was performed based on the pacing modality of the control group. The included studies were stratified into a “traditional pacing” subgroup (*n* = 3; including RVP, HBP, DDDR, and DDD-TPM) and a “physiological pacing” subgroup (*n* = 1; LBBAP). In the traditional pacing subgroup, heterogeneity was decreased (I^2^ = 43%), and no significant difference in adverse event risk was observed between leadless and traditional pacemakers. Conversely, the physiological pacing subgroup revealed a significantly higher risk of adverse events with leadless pacemakers compared to LBBAP (RR = 5.60, 95% CI: 1.51–20.72, *P* = 0.010). The test for subgroup differences was statistically significant (*P* = 0.04), identifying the control pacing modality as the primary source of heterogeneity. These findings suggest that the comparative safety profile of leadless pacemakers depends heavily on the control intervention, a factor that warrants careful consideration in clinical decision-making (Figure 3).

**Figure 3:**
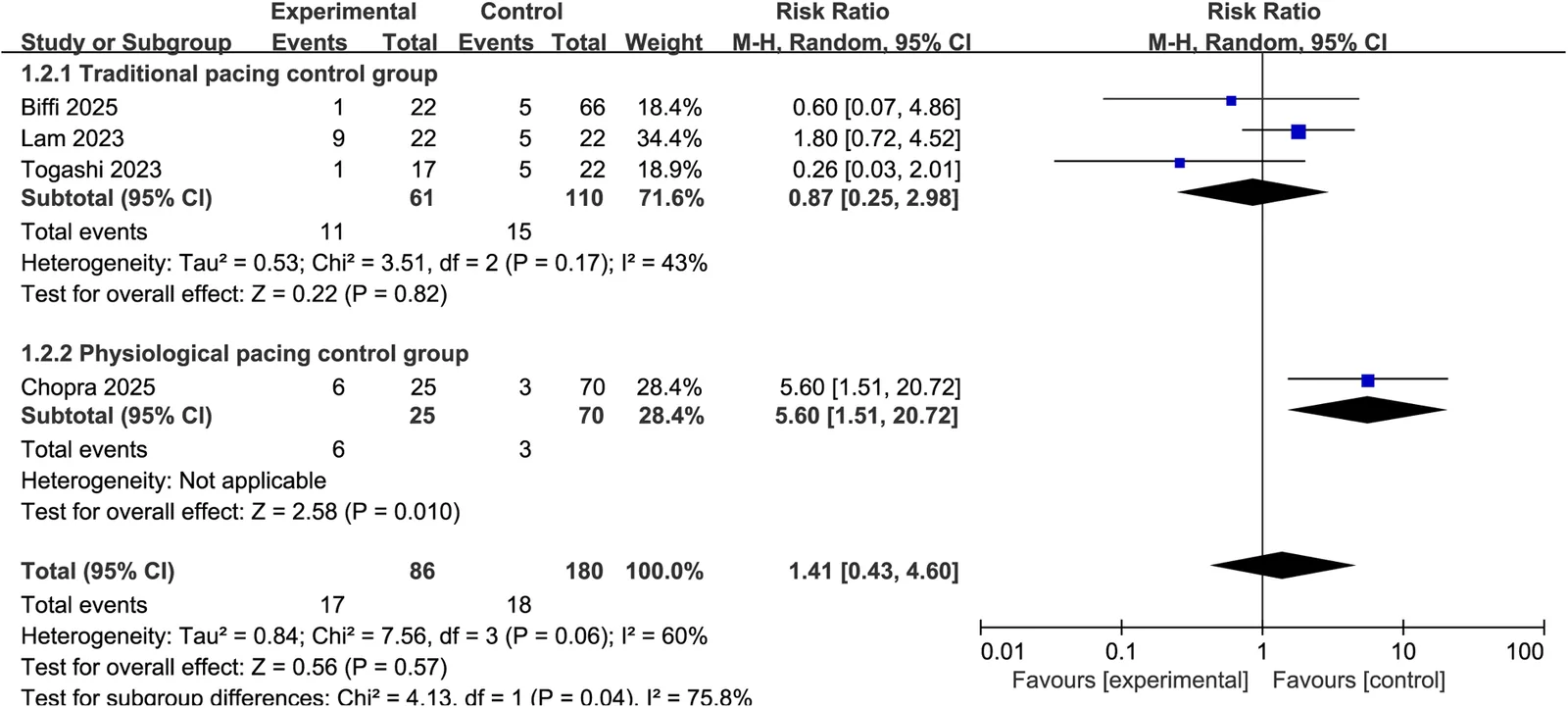
Subgroup analysis forest plot of adverse event risk for leadless pacemakers under different control group pacing modalities.

Secondly, considering the device heterogeneity inherent in leadless pacemakers, this study included literature for subgroup analysis based on device types. Among them, Togashi et al. (11) used a single-chamber ventricular pacing leadless pacemaker and classified it as the “Single-Chamber Ventricular Leadless Pacemaker” subgroup (*n* = 1); Biffi et al (8), Chopra et al. (10) and Lam et al (30) used atrioventricular synchronous/dual-chamber leadless pacemakers (Micra AV) and were classified as the “AV-Synchronous/Dual-Chamber Leadless Pacemaker” subgroup (*n* = 3). The subgroup analysis results showed that there was no significant difference in the risk of adverse events between the leadless pacemaker group and the control group in the single-chamber ventricular pacing leadless pacemaker subgroup (RR = 0.26, 95% CI: 0.03–2.01, *P* = 0.20), and there was also no significant difference in the risk of adverse events between the three groups in the atrioventricular synchronous/dual-chamber leadless pacemaker subgroup (RR = 2.16, 95% CI: 0.76–6.17, *P* = 0.15), and the difference between the three subgroups was not statistically significant, indicating that under current evidence, there is no significant difference in the risk of adverse events between different device types and traditional pacemakers (Figure 4).

**Figure 4:**
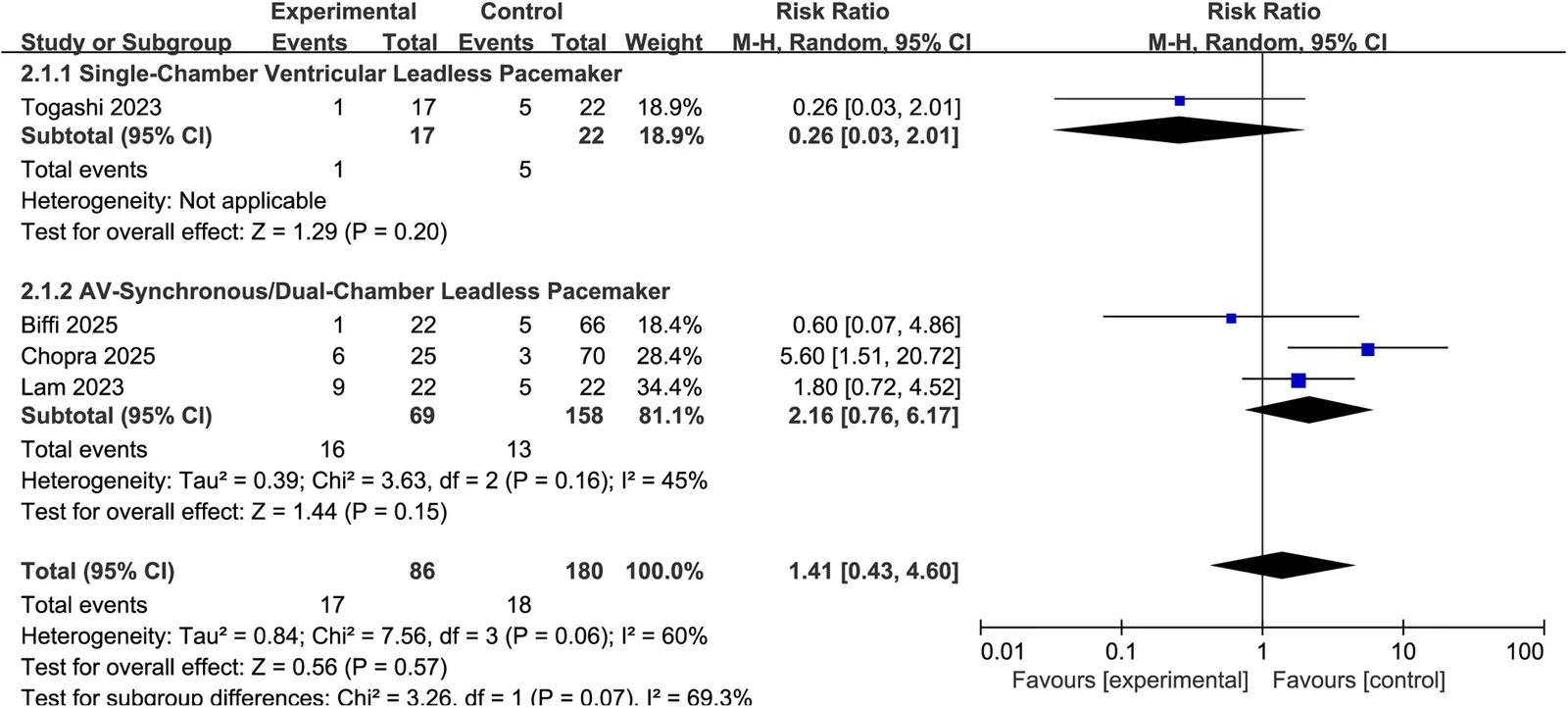
Forest plot of safety outcomes between different leadless pacemaker modes.

Another study conducted an internal control design to investigate the safety of different leadless pacemaker modes in AVB patients (9). Outcome measures included perioperative complications, chronic threshold elevation, pacing-induced cardiomyopathy, and pacemaker syndrome. This study enrolled 93 AVB patients, including 48 in the AV mode group with 5 adverse events and 45 in the VR mode group with 10 adverse events. Using a fixed-effects model, the analysis revealed a lower risk of adverse events in the AV mode group compared to the VR mode group (RR = 0.47, 95% CI: 0.17–1.27, *P* = 0.13). Although this difference was not statistically significant, it suggests a trend toward better safety with the AV mode in AVB patients. Given the small sample size, these findings warrant further validation in larger prospective studies (Figure 5).

**Figure 5:**
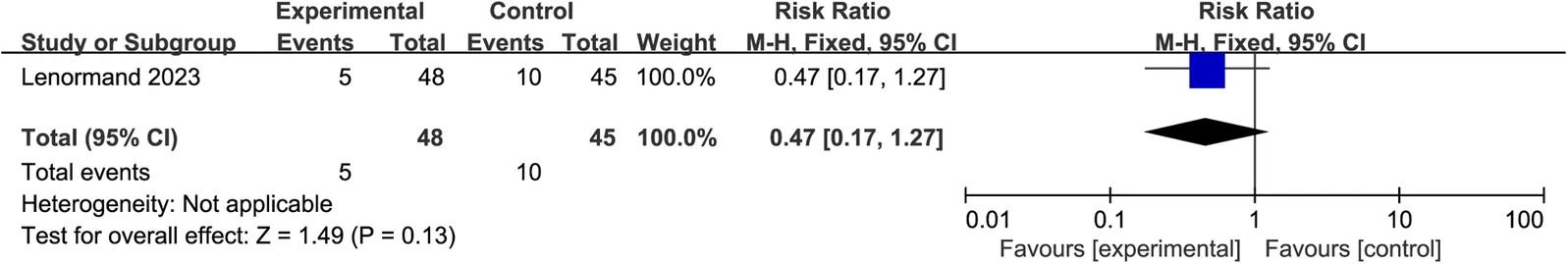
Comparison of adverse event risks between AV mode and VR mode leadless pacemakers in AVB patients.

#### 3.5.2 Single group rate analysis

A single-arm meta-analysis was conducted to pool the incidence of adverse events in patients with AVB treated with leadless pacemaker. Five cohort studies and eleven single-arm observational studies reporting adverse event rates in this population were included. Due to significant heterogeneity in study design, control settings, and baseline patient characteristics, overall data pooling was deemed inappropriate; instead, a stratified single-arm meta-analysis was conducted.

##### 3.5.2.1 Analysis of leadless pacemaker group in cohort studies

A single-arm meta-analysis of proportions was performed on the leadless pacemaker arms of five cohort studies (8–11, 30), encompassing 134 AVB patients and 22 adverse events. The forest plot demonstrated a concentrated distribution of effect sizes, and the pooled estimate highlights a consistently low event rate, further supporting the safety performance of the leadless pacemaker in these patients. The forest plot reveals substantial heterogeneity across the included studies (I^2^ = 70%, *P* = 0.010), with individual risk differences ranging widely from 0.05 to 0.41. Although the pooled risk difference of 0.14 (95% CI: 0.04 to 0.24, *P* = 0.005) suggests a statistically significant overall effect, the considerable between-study variability cautions against interpreting this as a uniformly low event rate. Therefore, while the leadless pacemaker demonstrates an overall favorable risk profile, its safety performance should be interpreted with caution due to the observed inconsistency among studies (Figure 6).

**Figure 6:**
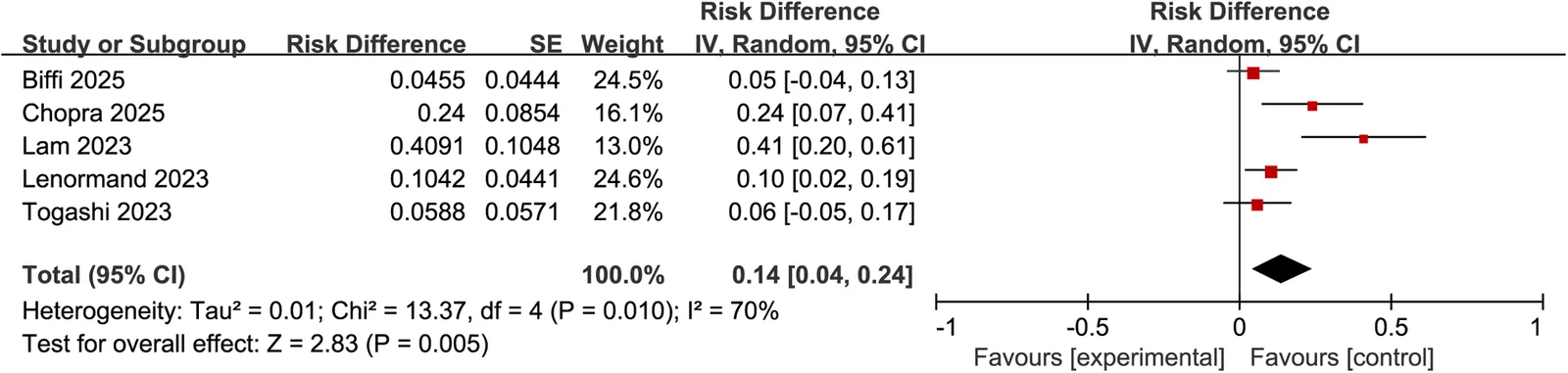
Forest plot of leadless pacemaker group in cohort studies.

##### 3.5.2.2 Analysis of single-arm studies

A total of 294 patients with AVB were included in 11 single-arm studies, and 1 adverse event occurred (21–24, 32, 51, 55, 58, 59, 64, 66). To address the zero event data and stabilize the variance of extreme event rates, the Freeman-Tukey double arcsine transformation was used prior to pooling via the inverse variance method. The heterogeneity test results showed no significant variance among the studies (I^2^ = 0.0%, *χ*^2^ = 1.87, *P* = 0.9973), prompting the use of a fixed-effects model (Figure 7). The pooled transformed effect size was 0.21 (95% CI: 0.10–0.33). Upon back-transformation, the overall incidence of adverse events was 1.15% (95% CI: 0.26%–2.67%). These findings demonstrate a consistently low adverse event rate for leadless pacemakers in these patients, indicating highly stable results.

**Figure 7:**
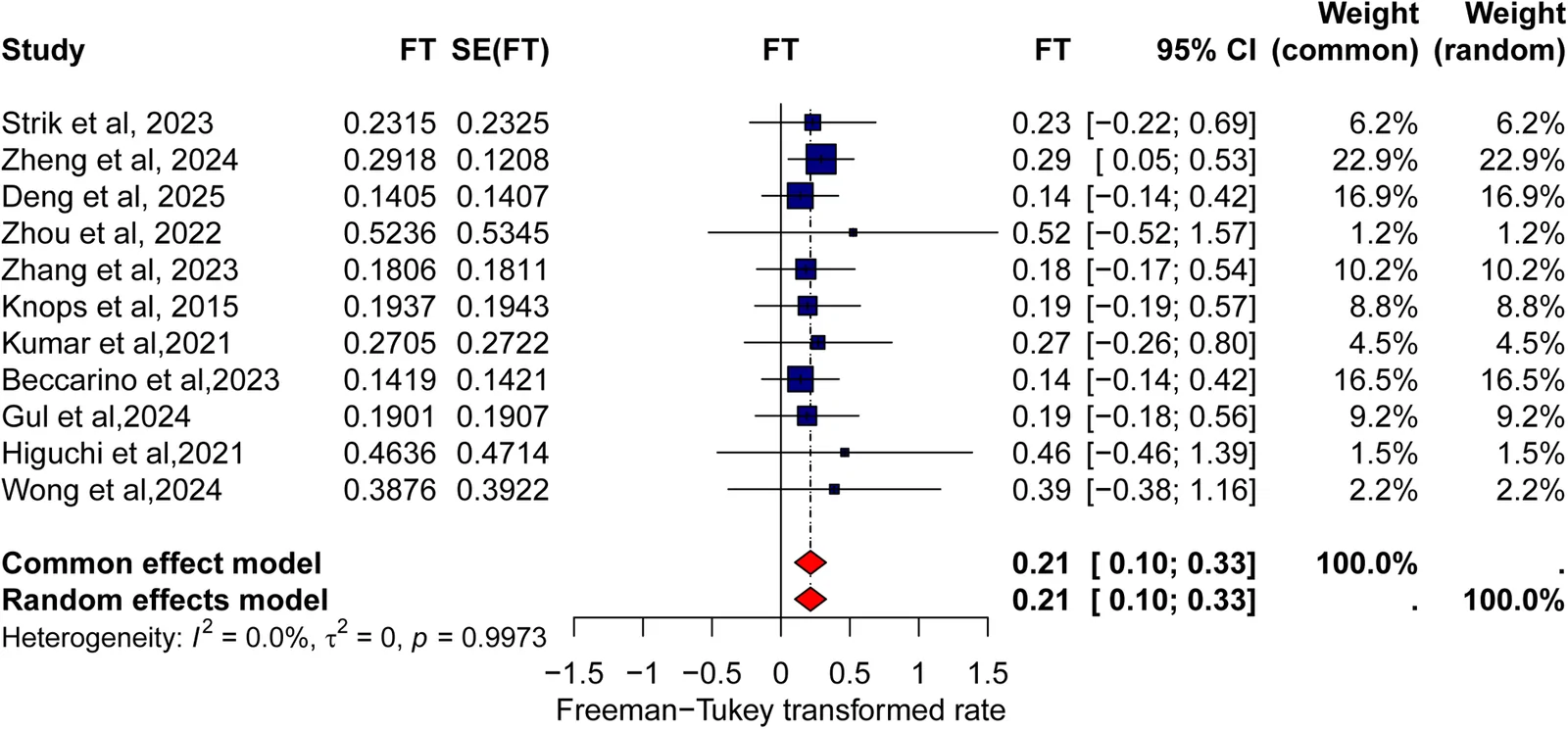
Forest plot of single-arm rate analysis (Freeman-Tukey double arcsine transformation).

Both analysis results suggest that leadless pacemakers possess an acceptable safety in patients with AVB. Synthesizing the findings from the two single-arm meta-analyses reveals that the incidence of adverse events remains low across both the single-arm observation population and the AVB subgroup of the cohort study. Furthermore,the heterogeneity between studies is within a low range, indicating good consistency in the results.

## 4 Discussion

In this systematic evaluation, leadless pacemakers demonstrated a safety profile comparable to traditional intravenous pacemakers. Despite the lack of a significant risk reduction, the consistently low incidence of adverse events supports the use of leadless pacing as a safe alternative for patients with AVB.

Descriptive analysis of 60 studies (*n* = 14,224) provides additional longitudinal support. The 60 studies included in this analysis covered the application of different types of leadless pacemaker devices in patients with AVB, with generally high implantation success rates across studies. Regarding adverse events, although there was some heterogeneity among the studies, the overall incidence was low and manageable. Taking the Micra series of leadless pacemakers as an example, multiple large-scale studies have confirmed their favorable safety profile. A retrospective analysis by Chen et al. of 1,620 patients who underwent Micra pacemaker implantation in the Shanghai region showed a total adverse event rate of 2.35%, with the incidence of major complications such as cardiac perforation, femoral vein thrombosis, and elevated pacing thresholds all below 1% (45). Garweg et al. followed 801 Micra AV implant recipients for 12 months and reported a major complication rate of 3.7%, with an incidence of pericardial effusion/perforation of only 1.2%; no device infections or dislodgements were reported, and there was a 58% reduction in major complication risk compared with conventional dual-chamber transvenous pacemakers (17). The Aveir DR series of leadless pacemakers has also demonstrated an acceptable safety profile in larger sample studies. Doshi et al. reported an overall complication rate of 11.1% among 452 patients, with very low incidences of serious complications such as pericardial effusion and cardiac perforation (50). Although some studies did not isolate AVB subgroup data, the high proportion of AVB indications in these large registries suggests that these favorable safety outcomes are highly generalizable to the AVB population.

In the comparative analysis, no statistically significant difference in safety was observed between the use of leadless pacemakers and traditional intravenous pacemakers in AVB patients (RR = 1.41, 95% CI: 0.43–4.60, *P* = 0.57), suggesting that the overall safety of the two pacing techniques is comparable in the AVB population. This finding diverges slightly from multiple previous meta-analyses, such as that by Gangannapalle et al., which reported a significantly lower risk of complications such as pneumothorax and endocarditis in the leadless group compared to traditional intravenous pacemakers (4). We hypothesize that this discrepancy stems from patient population heterogeneity. While previous studies included a wide range of patient populations, including atrial fibrillation with slow ventricular rate, syncope, heart failure, and other pacing indications. Our analysis focused exclusively on AVB. The specific physiological and anatomical considerations in AVB patients might mitigate some of the comparative advantages observed in more heterogenous cohorts. Furthermore, recent large-scale safety data further reinforce the anatomical advantage of leadless pacemakers. In a large consecutive case series, La Fazia et al. reported an extremely low incidence of new-onset severe tricuspid regurgitation (TR) after leadless pacemaker implantation, confirming that these devices exert substantially less mechanical interference on the tricuspid valve apparatus than conventional transvenous leads, thus mitigating the risk of hemodynamic compromise from valvular insufficiency (67). Taken together with our results, although overall major adverse events did not differ significantly in the AVB population, the favorable safety profile of leadless pacemakers in avoiding severe TR may render them particularly valuable in AVB subgroups with a heightened risk of subsequent valvular intervention.

Considering the inherent device heterogeneity among leadless pacemakers, we performed subgroup analyses stratified by device type. The results showed no statistically significant difference between subgroups, suggesting that, based on current evidence, neither device type demonstrated a significantly different risk of adverse events compared with conventional pacemakers. For patients with AVB, maintaining AV synchrony can significantly improve cardiac output and quality of life while avoiding pacemaker syndrome. In contrast, single-chamber pacing may only be suitable for patients with extremely low physical activity or those with persistent atrial fibrillation. Although our subgroup analysis did not detect a significant difference in adverse event risk between different device types and conventional pacemakers, it should be emphasized that equivalence in safety does not equate to equivalence in clinical effectiveness, which warrants further evaluation through specifically designed prospective studies.

Conventional transvenous pacemakers have progressively shifted toward conduction system pacing, such as His bundle pacing and left bundle branch area pacing, to achieve more physiological ventricular activation. However, contemporary leadless pacing systems remain confined to conventional right ventricular apical or septal pacing. This limitation is particularly relevant for patients with AVB, who generally require a high percentage of ventricular pacing. The dyssynchronous ventricular activation induced by conventional right ventricular pacing has been well recognized as a key factor contributing to pacing-induced cardiomyopathy and the subsequent development of heart failure during long-term follow-up.

The overall safety profile, as reflected by the incidence of adverse events in AVB, was further confirmed by our single-arm meta-analysis. We observed a low complication rate of 14% in cohort studies and a remarkably low 1.15% in single-arm studies. For instance, Biffi et al. reported a 4.5% complication rate among 22 AVB patients, which involved only one case of hematoma related to vascular puncture, with no other perioperative or long-term complications (8). Similarly, Togashi et al. demonstrated that in high-grade AVB patients, the incidence of adverse events remained low (6%) over a 2-year follow-up (11). The absence of severe perioperative complications across multiple studies further reinforces the clinical feasibility of leadless pacemakers in AVB patients.

The reasons for the certain degree of heterogeneity among the included studies in this meta-analysis may be as follows: Firstly, in the leadless group, the brands of pacemakers implanted in patients were not completely consistent, including Micra VR, Micra AV, VVI-LPM, etc., while in the traditional group, the brands of pacemakers implanted in patients were also inconsistent, resulting in bias. Secondly, in evaluating the complications caused by pacemakers, different studies use different ultrasound equipment and operators, which inevitably leads to bias. Then, the occurrence of postoperative complications is to some extent related to the surgical proficiency of the surgeon, which inevitably leads to bias due to the differences in surgeons in different studies. Lastly, inconsistent follow-up times between studies affect the comparability of results.

This study has the following limitations. Firstly, most original studies did not separately report complication data for the AVB subgroup, which precluded quantitative synthesis for this specific population. Secondly, we performed a subgroup analysis according to the type of leadless pacemaker where data were available. However, because the pacing modality in the transvenous pacemaker group was insufficiently reported, a corresponding subgroup comparison between conventional right ventricular pacing and physiological conduction system pacing could not be performed. Thirdly, access-site complications should also be interpreted with caution because implantation techniques differed between leadless and transvenous pacemaker systems. Transvenous pacemakers are typically implanted through the cephalic, subclavian, or axillary vein, whereas leadless pacemakers are delivered via the femoral vein. In addition, factors such as ultrasound-guided vs. landmark-guided venous access, operator experience, and vascular closure techniques may influence the incidence of hematoma and other vascular complications. Because these procedural details were not consistently reported in the included studies, their potential impact could not be evaluated in the present meta-analysis. Then, in evaluating the complications caused by pacemakers, different studies use different ultrasound equipment and operators, which inevitably leads to bias. Furthermore, the occurrence of postoperative complications is to some extent related to the surgical proficiency of the surgeon, which inevitably leads to bias due to the differences in surgeons in different studies. Lastly, inconsistent follow-up times between studies affect the comparability of results. In the future, more high-quality, large sample prospective studies should be conducted on the AVB subgroup to further validate the safety of leadless pacemakers in patients with different AVB subtypes and clinical backgrounds.

## 5 Conclusions

This study systematically evaluated the safety of using leadless pacemakers in patients with AVB. The existing evidence indicates that the risk of adverse events in this population is not significantly different from that of traditional intravenous pacemakers, suggesting that their safety is comparable. Single group rate analysis further showed that the overall incidence of complications in AVB patients with leadless pacemakers was low. A single-arm studies was further observed remarkably low. Descriptive analysis based on the overall population supports the feasibility and safety of its application in AVB patients. However, due to insufficient reporting of original research data, more high-quality, large sample studies targeting the AVB subgroup are still needed in the future to further validate the safety and applicability of leadless pacemakers in patients with different AVB subtypes and clinical backgrounds, providing more reliable evidence-based support for individualized device selection in clinical practice.
