# Supplementary material for: Leadless pacemaker in atrioventricular block patients with specific indications: a systematic review
Source: Front Cardiovasc Med. 2026 Sep 14;13:1910998. doi: 10.3389/fcvm.2026.1910998 (PMC13617243; doi:10.3389/fcvm.2026.1910998)
Supplement: Supplementary file 1 [file Table1.docx]

**Supplementary Table 1.** Literature Search Strategy

| **Database** | **Searchstrategy** |
| --- | --- |
| PubMed | ((Atrioventricular Block[MeSH Terms]) OR ((((atrioventricular block*[Title/Abstract]) OR (AV block*[Title/Abstract])) OR (complete heart block[Title/Abstract])) OR (third degree block*[Title/Abstract]))) AND (((((Leadless Pacemaker[Title/Abstract]) OR (Micra[Title/Abstract])) OR (Nanostim[Title/Abstract])) OR (leadless cardiac pacemaker[Title/Abstract])) OR (leadless pacing[Title/Abstract])) |
| Embase (via Elsevier) | ('cardiac pacemaker'/exp OR 'leadless pacemaker':ti,ab OR 'leadless pacing':ti,ab OR 'leadless cardiac pacemaker':ti,ab OR micra:ti,ab OR nanostim:ti,ab) AND ('atrioventricular block'/exp OR 'atrioventricular block':ti,ab OR 'atrioventricular conduction block':ti,ab OR 'AV block':ti,ab OR AVB:ti,ab) |
| Web of Science Core Collection | (TS=(leadless pacemaker* OR leadless cardiac pacemaker* OR leadless pacing OR wireless pacemaker* OR intracardiac pacemaker* OR Micra OR Nanostim OR lead-free pacemaker*)) AND TS=(atrioventricular block* OR AV block* OR AVB OR heart block* OR second degree block* OR third degree block* OR complete heart block OR high grade AV block OR atrioventricular conduction block) |
| Cochrane Library (CENTRAL) | ("leadless pacemaker" OR "leadless pacing" OR "leadless cardiac pacemaker" OR Micra OR Nanostim) AND ("atrioventricular block" OR "atrioventricular conduction block" OR "AV block" OR AVB) |
| China National Knowledge Infrastructure (CNKI) | （主题：无导线起搏器 + 无导线心脏起搏 + 无导线起搏 + 无导线起搏技术 + Micra + Nanostim）AND（主题：房室传导阻滞 + 房室阻滞 + 完全性房室传导阻滞 + AVB） |
| Wanfang Database | 主题:(无导线起搏器 OR Micra OR Nanostim) and 主题:(房室传导阻滞 OR 房室阻滞 OR 完全性房室传导阻滞 OR AVB) |
| ClinicalTrials.gov | Condition/disease: "Atrioventricular Block"; Intervention/treatment: ("leadless pacemaker" OR "leadless pacing" OR Micra OR Nanostim) |
| WHO International Clinical Trials Registry Platform (ICTRP) | ("atrioventricular block" OR "atrioventricular conduction block" OR "AV block" OR AVB) AND ("leadless pacemaker" OR "leadless pacing" OR Micra OR Nanostim) |
