# Supplementary material for: Leadless pacemaker in atrioventricular block patients with specific indications: a systematic review
Source: Front Cardiovasc Med. 2026 Sep 14;13:1910998. doi: 10.3389/fcvm.2026.1910998 (PMC13617243; doi:10.3389/fcvm.2026.1910998)
Supplement: Supplementary file 2 [file Table2.docx]

**Supplementary Table 2.** Quality assessment results of included cohort studies

| **study** | **Selection** | **Comparability** | **Outcome** | **score** |
| --- | --- | --- | --- | --- |
|  |  |  |  |  |
| Biffi et al., 2025 | ★★★★ | ★ | ★★★ | 8 |
| Katsuki et al., 2022 | ★★★★ | ★★ | ★★ | 8 |
| Sasaki et al., 2022 | ★★★★ | ★★ | ★★ | 8 |
| Lenormand et al., 2023 | ★★★★ | ★ | ★★ | 7 |
| Markson et al., 2025 | ★★★★ | ★★ | ★★ | 8 |
| Chopra et al., 2025 | ★★★ | ★ | ★★ | 6 |
| Mararenko et al., 2023 | ★★★★ | ★ | ★★★ | 8 |
| Schiavone et al., 2023 | ★★★★ | ★ | ★★ | 7 |
| Garweg et al., 2024 | ★★★★ | ★ | ★★ | 7 |
| Russo et al., 2022 | ★★★★ | ★ | ★★ | 7 |
| Ma et al., 2025 | ★★★ | ★ | ★★★ | 7 |
| Fouad et al., 2025 | ★★★★ | ★ | ★★★ | 8 |
| Togashi et al., 2023 | ★★★ | ★ | ★★ | 6 |
| Palmisano et al,2025 | ★★★★ | ★★ | ★★★ | 9 |
| Garweg et al, 2020 | ★★★★ | ★ | ★★ | 7 |
| Gao et al,2023 | ★★★★ | ★ | ★★ | 7 |
| Ze et al,2022 | ★★★★ | ★ | ★★ | 7 |
| Wang et al,2026 | ★★★★ | ★ | ★★ | 7 |
| Lam et al,2023 | ★★★★ | ★ | ★★ | 7 |
| Jelisejevas et al,2024 | ★★★★ | ★ | ★★ | 7 |

NOS, Newcastle-Ottawa Quality Assessment Scale; ★, when a certain condition is met, one star is awarded.

**Supplementary Table 3.** Quality assessment results of included single-arm studies

| **study** | **Q1** | **Q2** | **Q3** | **Q4** | **Q5** | **Q6** | **Q7** | **Q8** | **Q9** | **Q10** |
| --- | --- | --- | --- | --- | --- | --- | --- | --- | --- | --- |
| Sharma et al., 2020 | **√** | **√** | **√** | **√** |  | **√** | **√** | **√** |  | **√** |
| Arps et al., 2023 | **√** | **√** | **√** | **√** |  | **√** | **√** | **√** |  | **√** |
| Haddai et al., 2025 | **√** | **√** | **√** | **√** |  | **√** | **√** | **√** |  | **√** |
| Knops et al., 2023 | **√** | **√** | **√** | **√** |  | **√** | **√** | **√** |  | **√** |
| Ritter et al., 2015 | **√** | **√** | **√** | **√** |  | **√** | **√** | **√** |  | **√** |
| Yan et al., 2024 | **√** | **√** | **√** | **√** |  | **√** | **√** | **√** |  | **√** |
| Strik et al., 2023 | **√** | **√** | **√** | **√** |  | **√** | **√** | **√** |  | **√** |
| Zheng et al., 202 | **√** | **√** | **√** | **√** |  | **√** | **√** | **√** |  | **√** |
| Deng et al., 2025 | **√** | **√** | **√** | **√** |  | **√** | **√** | **√** |  | **√** |
| Zhou et al., 2022 | **√** | **√** | **√** | **√** |  | **√** | **√** | **√** |  | **√** |
| Garweg et al., 2017 | **√** | **√** | **√** | **√** |  | **√** | **√** | **√** |  | **√** |
| Knops et al., 2025 | **√** | **√** | **√** | **√** |  | **√** | **√** | **√** |  | **√** |
| Reddy et al., 2014 | **√** | **√** | **√** | **√** |  | **√** | **√** | **√** |  | **√** |
| Da Costa et al., 2016 | **√** | **√** | **√** | **√** |  | **√** | **√** | **√** |  | **√** |
| He et al., 2023 | **√** | **√** | **√** | **√** |  | **√** | **√** | **√** |  | **√** |
| Li et al., 2021 | **√** | **√** | **√** | **√** |  | **√** | **√** | **√** |  | **√** |
| Wu et al., 2023 | **√** | **√** | **√** | **√** |  | **√** | **√** | **√** |  | **√** |
| Chen et al., 2024 | **√** | **√** | **√** | **√** |  | **√** | **√** | **√** |  | **√** |
| Chen et al., 2025 | **√** | **√** | **√** | **√** |  | **√** | **√** | **√** |  | **√** |
| Liu et al., 2025 | **√** | **√** | **√** | **√** |  | **√** | **√** | **√** |  | **√** |
| Zhang et al., 2023 | **√** | **√** | **√** | **√** |  | **√** | **√** | **√** |  | **√** |
| Kuang et al., 2021 | **√** | **√** | **√** | **√** |  | **√** | **√** | **√** |  | **√** |
| Chinitz et al., 2023 | **√** | **√** | **√** | **√** |  | **√** | **√** | **√** |  | **√** |
| Doshi et al., 2025 | **√** | **√** | **√** | **√** |  | **√** | **√** | **√** |  | **√** |
| Knops et al, 2015 | **√** | **√** | **√** | **√** |  | **√** | **√** | **√** |  | **√** |
| Reddy et al, 2015 | **√** | **√** | **√** | **√** |  | **√** | **√** | **√** |  | **√** |
| Garweg et al, 2019 | **√** | **√** | **√** | **√** |  | **√** | **√** | **√** |  | **√** |
| Valiton et al,2019 | **√** | **√** | **√** | **√** |  | **√** | **√** | **√** |  | **√** |
| Kumar et al,2021 | **√** | **√** | **√** | **√** |  | **√** | **√** | **√** |  | **√** |
| Bicong et al,2022 | **√** | **√** | **√** | **√** |  | **√** | **√** | **√** |  | **√** |
| Mechulan et al,2022 | **√** | **√** | **√** | **√** |  | **√** | **√** | **√** |  | **√** |
| Beccarino et al,2023 | **√** | **√** | **√** | **√** |  | **√** | **√** | **√** |  | **√** |
| Gul et al,2024 | **√** | **√** | **√** | **√** |  | **√** | **√** | **√** |  | **√** |
| Oates et al,2024 | **√** | **√** | **√** | **√** |  | **√** | **√** | **√** |  | **√** |
| Sultan et al,2024 | **√** | **√** | **√** | **√** |  | **√** | **√** | **√** |  | **√** |
| El-Bokl et al,2022 | **√** | **√** | **√** | **√** |  | **√** | **√** | **√** |  | **√** |
| Denman et al,2018 | **√** | **√** | **√** | **√** |  | **√** | **√** | **√** |  | **√** |
| Higuchi et al,2021 | **√** | **√** | **√** | **√** |  | **√** | **√** | **√** |  | **√** |
| He et al,2022 | **√** | **√** | **√** | **√** |  | **√** | **√** | **√** |  | **√** |
| Wong et al,2024 | **√** | **√** | **√** | **√** |  | **√** | **√** | **√** |  | **√** |
